# Supplementary material for: Genome and transcriptome analyses reveal genes involved in the formation of fine ridges on petal epidermal cells in Hibiscus trionum
Source: DNA Res. 2023 Sep 11;30(5):dsad019. doi: 10.1093/dnares/dsad019 (PMC10558197; doi:10.1093/dnares/dsad019)
Supplement: dsad019_suppl_Supplementary_Figures_S1-S4 [file dsad019_suppl_supplementary_figures_s1-s4.pdf]

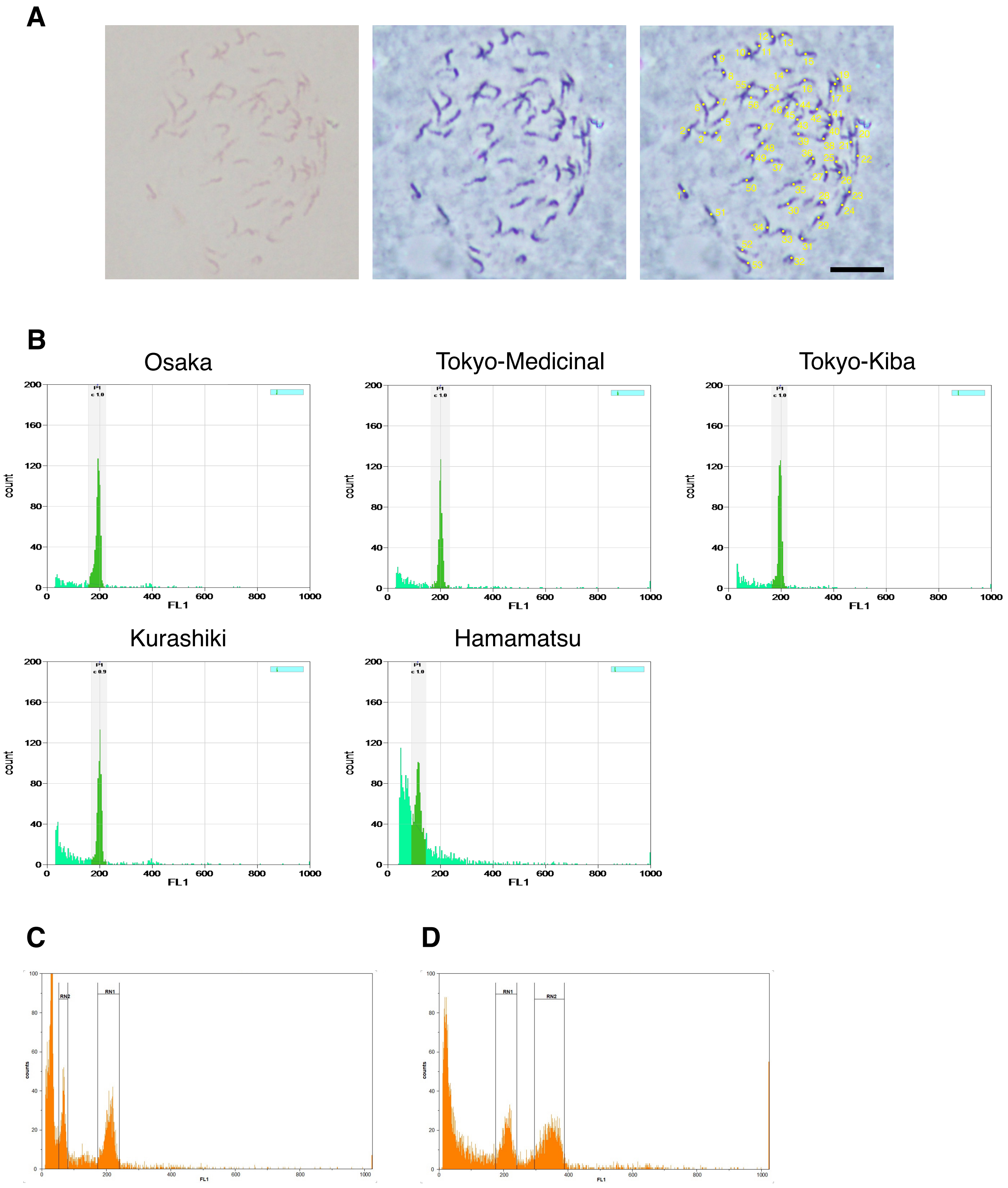

Supplementary Figure S1. Ploidy and genome size estimation in *H. trionum*. (A) Karyotype analysis in prophase using the Osaka line of *H. trionum*, showing 56 chromosomes. Yellow dots with numbers represent individual chromosomes. Scale bar = 10 μm. (B) Ploidy analysis with DAPI fluorescence signals by flow cytometry in each *H. trionum* line. Areas with gray backgrounds in each histogram indicate the peak positions. (C, D) Genome size estimation of the diploid *H. trionum* (Hamamatsu hibiscus) through flow cytometry. RN1 with a peak of 67.33 and RN2 with a peak of 209.13 in C are Hamamatsu hibiscus and *A. thaliana* (8C = 540 Mb), respectively. RN1 with a peak of 210.43 and RN2 with a peak of 344.94 in D are Hamamatsu hibiscus and *N. benthamiana* (about 3 Gb).

```
[M::ha_assemble] # bases: 35533428268; # corrected bases: 336012; # recorrected bases: 10585
[M::ha_assemble] size of buffer: 14.555GB
[M::ha_pt_gen::24472.431*30.01] ==> counted 38896553 distinct minimizer k-mers
[M::ha_pt_gen] count[4095] = 0 (for sanity check)
[M::ha_analyze_count] lowest: count[5] = 19742
[M::ha_analyze_count] highest: count[20] = 2873506
[M::ha_hist_line]     1: ****************** 514727
[M::ha_hist_line]     2: ** 49464
[M::ha_hist_line]     3: * 23480
[M::ha_hist_line]     4: * 18151
[M::ha_hist_line]     5: * 19742
[M::ha_hist_line]     6: * 28413
[M::ha_hist_line]     7: ** 47180
[M::ha_hist_line]     8: *** 81486
[M::ha_hist_line]     9: ***** 144505
[M::ha_hist_line]    10: ******** 239349
[M::ha_hist_line]    11: ************** 388271
[M::ha_hist_line]    12: ********************* 592942
[M::ha_hist_line]    13: ****************************** 861459
[M::ha_hist_line]    14: ***************************************** 1185119
[M::ha_hist_line]    15: ****************************************************** 1554323
[M::ha_hist_line]    16: ******************************************************************* 1930235
[M::ha_hist_line]    17: ******************************************************************************* 2281652
[M::ha_hist_line]    18: ****************************************************************************************** 2595073
[M::ha_hist_line]    19: ************************************************************************************************** 2807446
[M::ha_hist_line]    20: **************************************************************************************************** 2873506
[M::ha_hist_line]    21: ************************************************************************************************** 2822423
[M::ha_hist_line]    22: ********************************************************************************************* 2667558
[M::ha_hist_line]    23: ************************************************************************************ 2407461
[M::ha_hist_line]    24: ************************************************************************* 2097323
[M::ha_hist_line]    25: ************************************************************* 1755173
[M::ha_hist_line]    26: ************************************************** 1427649
[M::ha_hist_line]    27: *************************************** 1125088
[M::ha_hist_line]    28: ****************************** 852120
[M::ha_hist_line]    29: ********************** 638565
[M::ha_hist_line]    30: **************** 473403
[M::ha_hist_line]    31: ************ 350741
[M::ha_hist_line]    32: ********* 266184
[M::ha_hist_line]    33: ******* 206064
[M::ha_hist_line]    34: ****** 172057
[M::ha_hist_line]    35: ***** 150882
[M::ha_hist_line]    36: ***** 143370
[M::ha_hist_line]    37: ***** 139338
[M::ha_hist_line]    38: ***** 138930
[M::ha_hist_line]    39: ***** 142124
[M::ha_hist_line]    40: ***** 144197
[M::ha_hist_line]    41: ***** 142697
[M::ha_hist_line]    42: ***** 140337
[M::ha_hist_line]    43: ***** 134630
[M::ha_hist_line]    44: **** 127937
[M::ha_hist_line]    45: **** 119637
[M::ha_hist_line]    46: **** 110761
[M::ha_hist_line]    47: **** 101481
[M::ha_hist_line]    48: *** 92879
[M::ha_hist_line]    49: *** 83459
[M::ha_hist_line]    50: *** 76588
[M::ha_hist_line]    51: ** 68897
[M::ha_hist_line]    52: ** 62652
[M::ha_hist_line]    53: ** 58136
[M::ha_hist_line]    54: ** 53982
[M::ha_hist_line]    55: ** 51826
[M::ha_hist_line]    56: ** 49779
[M::ha_hist_line]    57: ** 47549
[M::ha_hist_line]    58: ** 46414
[M::ha_hist_line]    59: ** 44199
[M::ha_hist_line]    60: ** 44096
[M::ha_hist_line]    61: * 42827
[M::ha_hist_line]    62: * 41980
[M::ha_hist_line]    63: * 40339
[M::ha_hist_line]    64: * 39430
[M::ha_hist_line]    65: * 37896
[M::ha_hist_line]    66: * 36565
[M::ha_hist_line]    67: * 35451
[M::ha_hist_line]    68: * 33195
[M::ha_hist_line]    69: * 31194
[M::ha_hist_line]    70: * 29298
[M::ha_hist_line]    71: * 28584
[M::ha_hist_line]    72: * 26657
[M::ha_hist_line]    73: * 25466
[M::ha_hist_line]    74: * 23565
[M::ha_hist_line]    75: * 23080
[M::ha_hist_line]    76: * 22132
[M::ha_hist_line]    77: * 20931
[M::ha_hist_line]    78: * 20320
[M::ha_hist_line]    79: * 19911
[M::ha_hist_line]    80: * 19370
[M::ha_hist_line]    81: * 18672
[M::ha_hist_line]    82: * 18423
[M::ha_hist_line]    83: * 17520
[M::ha_hist_line]    84: * 17366
[M::ha_hist_line]    85: * 16685
[M::ha_hist_line]    86: * 16027
[M::ha_hist_line]    87: * 15453
[M::ha_hist_line]    88: * 14887
[M::ha_hist_line]  rest: ***** 148220
```

Peak 1

Peak 2

Supplementary Figure S2. K-mer frequency plot from Hifiasm assembler. Orange bars indicate peak positions, Peak 1 and 2.

SHN family genes

htrionum.r.g51082 (expressed)

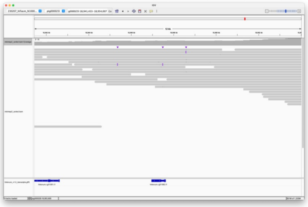

htrionum.r.g33629

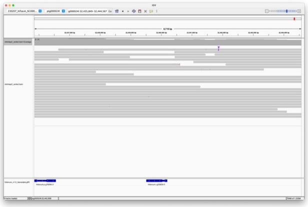

htrionum.r.g62214

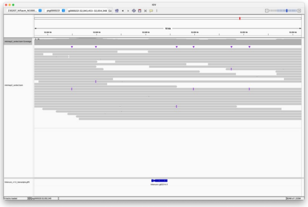

htrionum.r.g63066

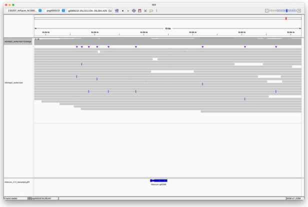

htrionum.r.g63489

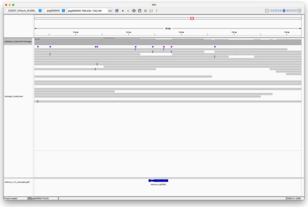

htrionum.r.g65607

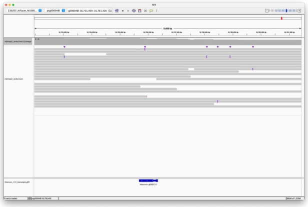

htrionum.r.g72950

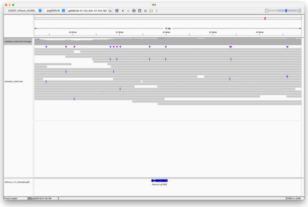

htrionum.r.g73448

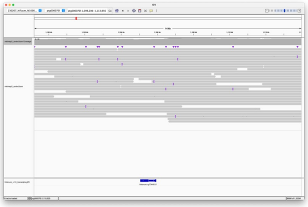

htrionum.r.g82579

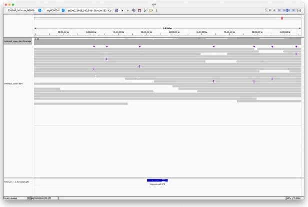

htrionum.r.g82775

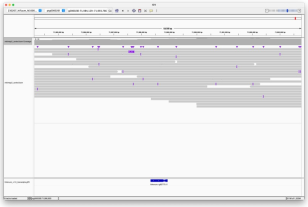

CUS2 genes

htrionum.r.g2803

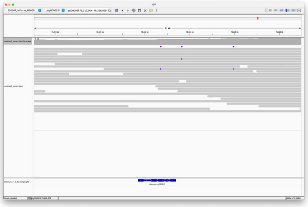

htrionum.r.g8620

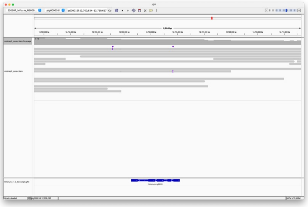

Genes involved in cutin synthesis

htrionum.r.g77092

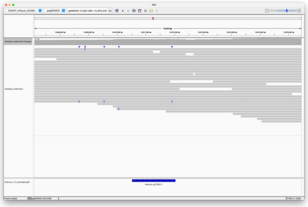

htrionum.r.g41935

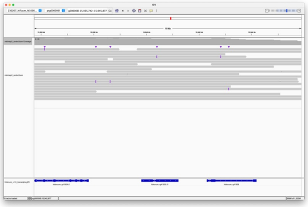

htrionum.r.g50765

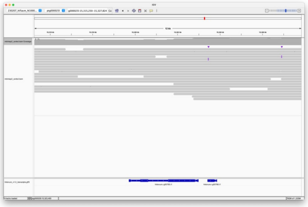

Supplementary Figure S3. Visualization of the mapping status of Hifi reads. The screen capture images of IGV in each gene locus are displayed. Indels, mismatches and read coverage are shown in each screen capture image.

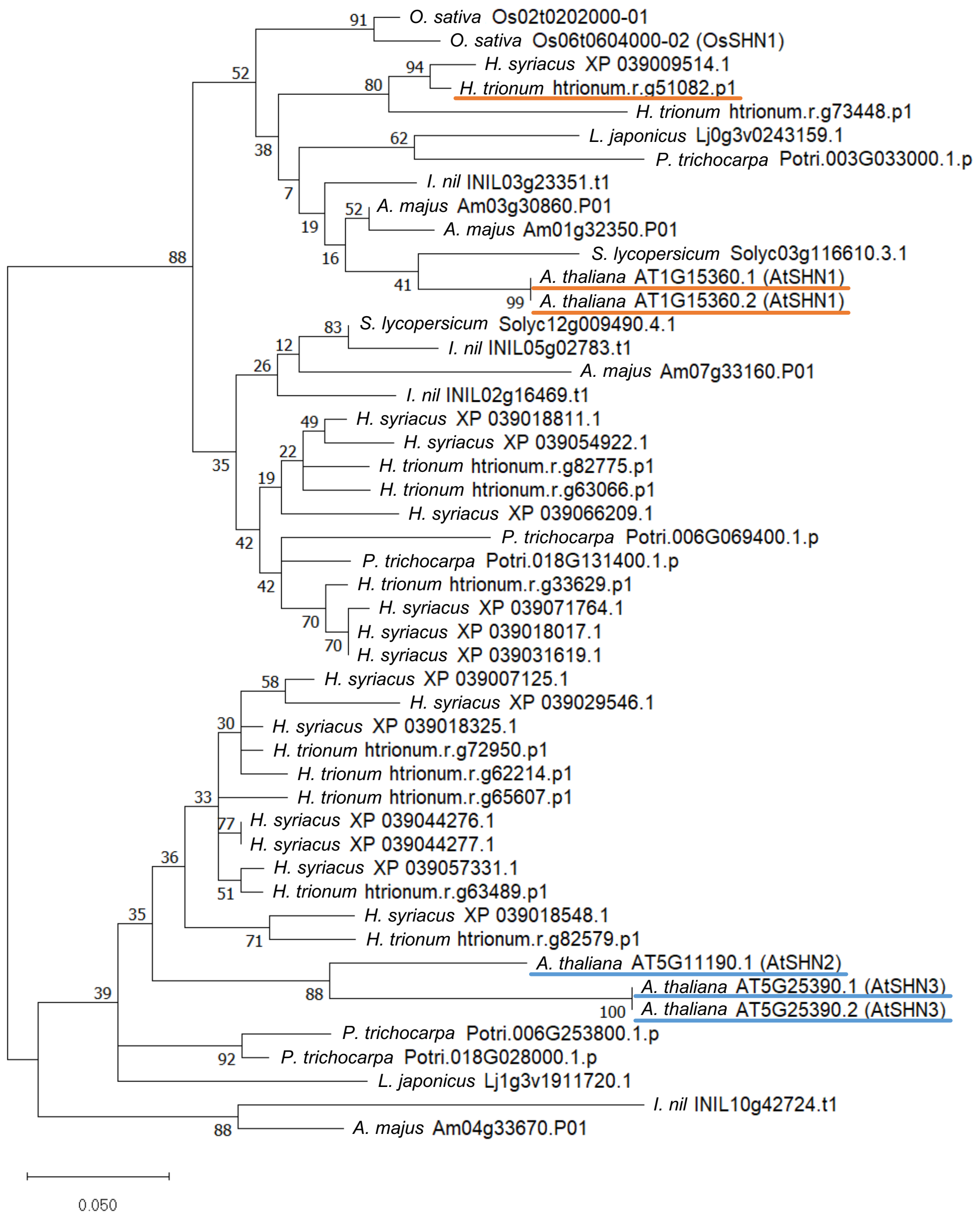

Supplementary Figure S4. Phylogenetic tree of the SHINE clade proteins. Branch lengths are proportional to the estimated number of amino acid substitutions per site (scale at lower left). The values shown at the nodes of the branches are bootstrap support values.
